# Equivalence and switching between biosimilars and reference molecules in rheumatoid arthritis: protocol for a systematic review and meta-analysis

**DOI:** 10.1186/s13643-021-01754-x

**Published:** 2021-07-17

**Authors:** Bruna O. Ascef, Matheus O. Almeida, Ana Cristina de Medeiros Ribeiro, Danieli C. O. Andrade, Haliton A. de Oliveira Júnior, Tiago V. Pereira, Patrícia C. de Soárez

**Affiliations:** 1 Programa de Pós-Graduação em Saúde Coletiva, Departamento de Medicina Preventiva, Faculdade de Medicina - FMUSP, Universidade de São Paulo, São Paulo, SP Brazil; 2 Programa de Pós-Graduação em Fisioterapia, Universidade Ibirapuera, São Paulo, SP Brazil; 3 Disciplina de Reumatologia do Hospital das Clínicas da Faculdade de Medicina, Universidade de São Paulo, São Paulo, SP Brazil; 4 Programa de Pós-Graduação em Medicamentos e Assistência Farmacêutica, Departamento de Farmácia Social, Universidade Federal de Minas Gerais, Belo Horizonte, MG Brazil; 5 Applied Health Research Centre, Li Ka Shing Knowledge Institute, St Michael’s Hospital, Toronto, ON Canada; 6 Department of Health Sciences, College of Medicine, University of Leicester, Leicester, UK

**Keywords:** Biosimilar pharmaceuticals, Etanercept, Infliximab, Adalimumab, Arthritis, Rheumatoid

## Abstract

**Background:**

Biologic drugs such as adalimumab, etanercept, and infliximab represent major first-line and second-line treatments for rheumatoid arthritis (RA) patients. However, their high cost poses a massive burden on healthcare systems worldwide. The expiration of patents for these biologics has driven the production of biosimilar drugs, which are potentially less costly and remarkably similar, albeit not identical to the reference molecules. This paper aims to outline the protocol of a systematic review that will investigate the efficacy and safety profile of biosimilars compared to biologics (objective 1) and the impact of switching between biosimilar drugs and reference biologics on the management of RA patients (objective 2).

**Methods:**

We will investigate the effects of any biosimilars of adalimumab, etanercept, and infliximab on RA patients. We will include randomized controlled trials (RCTs) or quasi-RCTs to assess efficacy and safety outcomes and RCTs with two- or multiple-part designs to evaluate the consequences of switching from reference biologics to biosimilar drugs (and vice-versa). Electronic searches will be performed through MEDLINE (via PubMed), EMBASE, LILACS, and CENTRAL (from inception to April 2021). Two independent reviewers will screen studies, extract data, and evaluate the risk of bias. The latter will be carried out considering specific domains from equivalence trials and switching studies. Random-effects models will be fitted to obtain summary estimates using either relative risk or standardized mean difference as a metric. The primary outcome will be the rate of treatment success according to the American College of Rheumatology 20 (ACR20), and the co-primary outcome will be the Health Assessment Questionnaire—Disability Index (HAQ-DI). Conclusions will be based on equivalence hypothesis testing using predefined margins of equivalence elicited from a group of experienced rheumatologists and prior studies. The overall certainty of the evidence will be assessed based on the GRADE system.

**Discussion:**

The present investigation proposes a comprehensive, clinician-oriented approach to assess the equivalence and the impact of switching between biosimilars and biologics on the management of patients with RA. Our results will elucidate the efficacy, safety, immunogenicity of biosimilars, and the clinical consequences of substituting biologics with biosimilars in the management of RA.

**Systematic review registration:**

PROSPERO CRD42019137152 and CRD42019137155

**Supplementary Information:**

The online version contains supplementary material available at 10.1186/s13643-021-01754-x.

## Background

Rheumatoid arthritis (RA) is a chronic inflammatory joint disease that affects up to 20 million people worldwide, thereby representing a major public health burden with important socioeconomic consequences [1–3].

Biological drugs, commonly known as “biologics,” are invaluable resources in the treatment of RA patients. Synthetic disease-modifying antirheumatic drugs (DMARDs) are the first line of therapy and associated with biologic DMARDs have changed clinical outcomes, reducing the inflammatory burden of disease and, therefore, chronic articular deterioration [4]. The effectiveness and safety of biologic DMARDs have been robustly established [5–7], and several studies have identified factors that affect the patient’s response to these DMARDs [8–11]. However, biologics pose an important challenge for the sustainability of healthcare systems worldwide, given the high direct costs associated with this drug category [1]. For instance, expenses related to biologic treatments can represent almost 40% of the net drug spending in the USA [12].

Given the rapid evolution of pharmaceutical technologies over the past decade and patent expiration of previously approved biologic molecules, biosimilar drugs have been developed as less costly alternatives to their reference biologics [13]. According to the US Food and Drug Administration (FDA), biosimilars possess clinically similar benefits and safety profile compared to the existing FDA-approved biologics [14]. In this regard, it is believed that biosimilars can accelerate the rheumatic disorder drug market competition, positively impacting the global healthcare system through improved healthcare affordability and increased patients’ access to effective and safe drugs [13, 15].

However, despite the cost-saving potential of biosimilar drugs, there are still diverging perceptions regarding the efficacy, safety, and immunogenicity of these follow-on biologics [16–18]. Importantly, the switching and interchangeability between biologic and biosimilar drugs are still topics of great debate in the treatment of RA [16–20].

Herein, we describe the protocol of a systematic review that will address the efficacy, safety, and immunogenicity of biosimilars compared to biologics, and the impact of switching between biosimilar drugs and reference biologics on the management of RA patients. Unlike previous reviews, we will establish acceptable equivalence margins elicited from clinical specialists to conclude on the equivalence of biosimilars compared to biologics.

## Methods/design

### Reporting guidelines used in this protocol

The present protocol followed the Preferred Reporting Items for Systematic Reviews and Meta-Analyses Protocols guidelines (PRISMA-P) 2015 statement (Additional file 1) [21]. We will refer separately to the main objectives of the systematic review as efficacy and safety (objective 1) and switching (objective 2) because they need varying methodologies and approaches.

### PROSPERO synopses

Synopses for the two main objectives were prospectively and separately registered in the International Prospective Register of Systematic Reviews (https://www.crd.york.ac.uk/PROSPERO/). The first objective will focus on the efficacy and safety of biosimilars compared to biologics (PROSPERO number: CRD42019137152), whereas the second objective will examine the clinical impact of switching from reference biologics to biosimilars on the management of RA patients whose treatment has already been started (PROSPERO: CRD42019137155).

### Adopted reporting and developing standards

The proposed systematic review will be reported following Preferred Reporting Items for Systematic Review and Meta-analysis (PRISMA Statement) [22]. We will also follow the guidelines of the Cochrane Handbook for conducting systematic reviews of interventions [23]. Besides, since there are specific aspects related to the conduct, interpretation, and reporting of equivalence and non-inferiority trials, we will also adopt the US Agency for Healthcare Research and Quality recommendations [24].

### Electronic searches

Search strategies were built using controlled vocabulary according to each database and free-text terms based on the research question. We will use the following electronic databases (from inception to April 2021): MEDLINE via PubMed, EMBASE, Cochrane Central Register of Controlled Trials (CENTRAL), and Latin American and Caribbean Health Science (LILACS). A detailed description of the search strategy is available in Additional file 2.

### Other sources

We will also search for non-published or ongoing trials in the EU Clinical Trial Register (https://www.clinicaltrialsregister.eu), International Clinical Trials Registry Platform-World Health Organization (http://apps.who.int/trialsearch/), and Clinicaltrials (https://clinicaltrials.gov/). The search strategies to be used in these platforms are described in Additional file 2. When necessary, we will contact corresponding authors for supplementary information. Additionally, we will manually screen the references of all included trials as well as previous systematic reviews. Finally, we will employ Google Scholar and Epistemonikos (https://www.epistemonikos.org/) to retrieve relevant reports citing all relevant included articles. No language limitation will be imposed.

### Eligibility criteria

#### Types of biosimilars

We will assess any biosimilars of adalimumab, etanercept, and infliximab. We chose these three main biologics because they are the most prescribed first-line biologic DMARDs in RA [25]. Also, these three DMARDS have the highest numbers of approved biosimilars for RA in the market [13, 25].

#### Types of control interventions

We will consider as control interventions the reference biologic drugs (i.e., adalimumab, etanercept, and infliximab originals). No restrictions on dosages, treatment schedules, co-treatment, or combined therapies will be imposed.

#### Types of trials

##### Types of trials: objective 1 (efficacy and safety)

To assess the efficacy and safety of biosimilars (“biosimilarity”) [27], we will include randomized controlled trials (RCTs) or quasi-RCTs. We will include all trials comparing biosimilars to biologic drugs irrespective of the type of statistical design (superiority, equivalence, or non-inferiority). A quasi-randomized trial was defined as a prospective interventional study whose allocation sequence was not truly random (e.g., consecutive order, day of the week, date of birth, etc.). For trials with a 2-part study design, we will consider results from the first period (biosimilarity) only to avoid carry-over effects.

##### Types of trials: objective 2 (switching)

To assess the impact of switching on clinical outcomes of RA patients, we will include RCTs with two- or multiple-part designs. The following four main designs of switching trials will be considered:

- Single-switch design [28, 29]: Trials in which there is a single switch from each treatment to the other. All patients receive the study interventions in successive periods. Firstly, patients are randomly allocated to either a biosimilar or a biologic drug (first period). Then, in the second period, treatments are randomly switched in both directions (group 1: biologic → biosimilar; group 2: biosimilar → biologic OR group 1: biologic → biosimilar, group 2: biosimilar → biologic; group 3: biologic → biologic; group 4: biosimilar → biosimilar).
- Transition design 1 (two non-switching groups as a control): Trials in which there is a single switch from one treatment (biologic drug) to another (biosimilar drug), but not the contrary. Firstly, patients are randomly allocated to either a biosimilar or a biologic drug (first period). Then, in the second period, the trial becomes a three-arm trial in which patients in the biologic drug group are re-randomized either to continue in the biologic group or to switch to the biosimilar drug treatment. Patients initially allocated to the biosimilar group continue to receive a biosimilar throughout the study period (experimental group: biologic → biosimilar; control arm 1: biologic → biologic; control arm 2: biosimilar → biosimilar).
- Transition design 2 (randomized trials with an open-label extension; single non-switching group as a control): Trials in which there is a single switch from a biologic drug to a biosimilar drug, but not the contrary. Firstly, patients are randomly allocated to either a biosimilar or a biologic drug (first period). Then, in open-label extended phase (second period), all patients (intervention and control groups) receive the biosimilar drug (experimental group: biologic → biosimilar; control arm 1: biosimilar → biosimilar).
- Multiple switches design: Also known as interchangeability design [28, 29], in which multiple switches between treatments are allowed throughout the trial follow-up.

#### Type of participants

Trials will be included if patients with RA had been diagnosed with validated and established international criteria. No limitation will be imposed on age, baseline RA severity, sex, lines of treatment (e.g., treatment-naïve patients or second line of treatment), or any other major demographic characteristics.

#### Types of outcome measures

All outcomes were prespecified in the registered PROSPERO synopses and were categorized into three types: efficacy (encompassing outcomes related to disease activity, functional capacity, quality of life, and structural damage progression), safety, and immunogenicity. For efficacy outcomes, we will extract data at the following time points: 1 month (± 2 weeks), 3 months (± 4 weeks), 6 months (± 4 weeks), 8 months (± 4 weeks), 12 months (± 4 weeks), 36 months (± 4 weeks), and 48 months (± 4 weeks). For safety and immunogenicity outcomes, we will collect data from longest follow-up available.

##### Primary outcomes

We prespecified a primary outcome, a co-primary outcome, and all secondary outcomes. A co-primary outcome was adopted because the demonstration of superiority or equivalence in a single outcome is insufficient to support clinical decisions for the management of RA patients. The choice of primary and co-primary outcomes was decided on a panel composed of two RA specialists supervised by two researchers with experience in evidence synthesis. The rationale was to evaluate the equivalence between biosimilars and reference biologic drugs using the minimum set of clinical outcomes that incorporate both physician-reported and patient-reported outcomes. Similar approaches have been used previously in RA trials [30, 31].

##### Objective 1: efficacy and safety

The primary outcome will be treatment success at 6 months according to the American College of Rheumatology 20 (ACR20) [32].

The co-primary outcome will be HAQ-DI, which assesses the functional status of patients through the evaluation of eight domains of daily life activities. The highest score reported for any component question in each domain determines the final score for that domain. By convention, the overall disability index is expressed on a 0 to 3 scale, representing an average score across the domains. A HAQ-DI of 0 indicates no functional disability, whereas a HAQ-DI of 3 denotes severe functional disability [33].

If trials report results at different time points, we will use the time point closest to 6 months.

##### Objective 2: the impact of switching

The primary outcome will be the rate of treatment success at 6 months after the first switch (i.e., 6 months after re-randomization or 6 months after the first switch on the open-label extension phase) defined by the ACR20 (dichotomous outcome). The co-primary outcome will be the HAQ-DI index also measured at 6 months after the first switch (continuous outcome). If outcome data are reported at different time points, we will use the time point closest to 6 months.

##### Secondary outcomes (efficacy, safety, and immunogenicity)

*Secondary outcomes: efficacy*

Secondary outcomes of efficacy will be examined at 6 months of follow-up (or the time point closest to 6 months) and will include disease activity, prevention of structural damage progression, and quality of life measures:

- Measures of disease activity: the American College of Rheumatology criteria with 50% (ACR50) and 70% (ACR70) responses, simplified disease activity score (SDAI), clinical disease activity score (CDAI), disease activity score in 28 joints based on the erythrocyte sedimentation rate (DAS28-ESR), disease activity score in 28 joints with four components based on C-reactive protein (DAS28-CRP), and the numeric index of the ACR response (ACR-N).
- Functional capacity/quality of life: scores of HAQ-DI and the Medical Outcomes Study 36-item Short-Form Health Survey (SF-36) (physical and mental components summaries).
- Prevention of structural damage progression: scores of Sharp/Van der Heijde or Sharp-Van Der Heidje Modified Score Method (mTRSS). A full description of secondary outcomes can be found in Additional file 3.

*Secondary outcomes: safety*

We will evaluate the safety of biosimilars compared to biologics by the proportion of patients with treatment-emergent adverse events (TEAEs), serious TEAEs, infusion-related reactions (IRRs), injection site reactions (ISRs), hypersensitivity, malignancies, active tuberculosis, serious infections, all-cause mortality, and treatment-related mortality. Also, we will evaluate discontinuation rates in both treatments. A full list of safety outcomes can be found in Additional file 3.

*Secondary outcomes: immunogenicity*

Immunogenicity will be evaluated by the proportion of patients with positive anti-drug antibodies (ADAs) and the proportion of patients with positive neutralizing antibodies (Nabs).

### Investigator training and data calibration

All investigators involved in the study selection, data extraction, and risk of bias assessments will be trained. Specifically, we will include a sample of four trials and perform calibration among reviewers, followed by oral and written instructions. We will perform multiple rounds of “calibration checks” throughout the data extraction process. The data extraction process will be guided by a codebook, which contains detailed technical information on each variable, definitions, assumptions, and possible responses (in case of categorical responses).

### Study screening and selection

We developed a customized web platform for data extraction and curation using Ragic (www.ragic.com). This database was carefully designed to simultaneously allow for study screening and selection and data extraction for the systematic review. Two independent investigators will perform all steps. Specifically, during the screening phase, two review authors will independently evaluate titles and abstracts. Disagreements will be solved by a consensus. Next, for each study selected, full-length articles will be downloaded, and two independent reviewers will re-assess the eligibility of each pre-selected trial. In cases of disagreements, a third reviewer will be consulted. Reasons for exclusions will be described in detail in subsequent publications.

### Data extraction and management

#### Analysis population

Trials may report two populations for the analysis: an intention-to-treat (ITT) population and a per-protocol (PP) population [24]. For the analysis of both objectives 1 and 2, preference will be given to results based on the PP population, because of the conservative effect of the per-protocol approach on equivalence testing [24]. Since there may be a substantial variety in the definition of what constitutes a PP population or an ITT analysis, we will collect and tabulate in detail the definition of PP and ITT used in each trial.

#### Numerical and graphical results

All data will be extracted independently by two investigators. Discrepancies will be solved via a consensus. We will extract all pertinent quantitative information, including the number of participants at baseline, the number of participants analyzed, and measures of central tendency, variability, and precision. Specifically, whenever available, we will collect means, mean changes, the difference between means at follow-up, medians, standard deviations, interquartile ranges, standard errors, confidence intervals (and their coverage, e.g., 90 or 95%), *P*-values (one- or two-sides), and t statistics. These data will be used to approximate means and standard deviations when necessary [34]. For continuous outcomes, we will use follow-up data preferentially but will use the mean change from baseline when follow-up values are not available [35].

Quantitative data from figures and graphs will be extracted independently using digitizing software (Digitizelt 2.2.2, Germany, https://www.digitizeit.de/). Estimates from the digitizing software will be averaged out to generate the final value. When necessary, data for the same trial will be extracted from multiple sources (e.g., multiple related publications and trial registries). Multiple publications from the same trial will be linked via a unique identifier. The linkage of several articles to the same identifier will be performed via the number assigned to the study’s registration (e.g., The National Clinical Trial number [NCT] from clinicaltrials.gov). When the trial registration number was not explicitly reported, we will perform a careful evaluation of the similarity between the eligible trial and those already included in the systematic review. The similarity will be rated by two reviewers based on the patient enrollment period, the affiliation of the trial investigators, participant institutions, target sample size, funding source, and patients’ baseline characteristics. In case of multiple publications reporting results for the same time point, we will extract data from the most complete or most recent article.

#### Ongoing trials

We will summarize all identified ongoing trials, detailing the primary author, research question(s), methods, outcome measures, and study start date, along with an estimate of the study completion date.

### Assessment of risk of bias

Two review authors will independently assess the risk of bias in the included studies. Each domain will be classified as being at a low, unclear, or high risk of bias. Disagreements will be resolved by consensus or discussion with a third reviewer. The studies will be assessed by outcome level. If the trial has one or more domains with a high risk of bias, it will be considered as a high risk of bias study. If the trial has more than two domains at uncertain risk of bias, we will judge the risk of bias to be uncertain. If the trial has a low risk of bias in all domains or one domain as uncertain bias, it will be considered as a low risk of bias study.

#### Assessment of risk of bias in efficacy and safety trials

We will use criteria recommended by the Cochrane collaboration (Cochrane Risk of bias tool 1.0) [36]. The following domains will be evaluated: random sequence generation, allocation concealment, blinding of participants and investigators, blinding of outcome assessors, and incomplete outcome data (PP and ITT population analysis). To specifically address equivalence or non-inferiority trials, we will refer to the recommendations by the US Agency for Healthcare Research and Quality [24] (Table 1). Specifically, we will assess the inconsistent application of inclusion/exclusion criteria, patients selected for anticipated nonresponse or good response in one arm, patient behavior changes (poor adherence, use of concomitant treatments, and protocol violations), inadequate outcome measurement techniques, and incomplete outcome data (PP and ITT population analysis: ITT population analysis may underestimate the treatment effect in equivalence/non-inferiority trials). More information on the criteria used in each domain can be found in Additional file 4.

**Table 1:** Risk of bias domains to be evaluated on equivalence or non-inferiority trials

Type of bias	Domain	Source
Selection bias	Random sequence generation	Cochrane RoB tool 1.0
	Allocation concealment	Cochrane RoB tool 1.0
	Inconsistent application of inclusion/exclusion criteria	US Agency for Healthcare Research and Quality
Performance bias	Blinding of participants and investigators	Cochrane RoB tool 1.0
	Participants behavior changes	US Agency for Healthcare Research and Quality
Detection bias	Blinding of outcome assessment	Cochrane RoB tool 1.0
	Outcome measurement techniques	US Agency for Healthcare Research and Quality
Attrition bias	Incomplete outcome data	Cochrane RoB tool 1.0 and US Agency for Healthcare Research and Quality

Sources: Cochrane Risk of Bias in randomized trials (RoB 1.0.) described in the *Cochrane Handbook for Systematic Reviews of Interventions* [36] and the US Agency for Healthcare Research and Quality recommendations [24]

#### Assessment of risk of bias in switching trials

For switching trials, we will use the recommendations of Moots et al. [28] and the FDA guidance for considerations in demonstrating interchangeability with a reference product [14]. The six specific domains to be evaluated are:

- Randomized and blinded design with appropriate control arms;
- At least 1-way switch from originator to biosimilar;
- The assessment of immunogenicity;
- The wash-out period between treatment;
- Enough power to assess efficacy and safety (equivalence phase); and
- Enough follow-up periods.

More information about the criteria of judgments can be seen in Additional file 5.

### Data synthesis

#### Effect size measures

For binary outcomes, we will combine study estimates using the relative risk (RR) as a measure of effect. For continuous outcomes, we will use the standardized mean difference (SMD) defined as the bias-adjusted method of Hedges. SMD will be used because it has similar statistical power and is more generalizable than the mean difference [37].

#### Meta-analysis models

Main analyses will be based on the random-effects model with the restricted maximum-likelihood estimator for the between-study variance [38]. A random-effects model was prespecified as the primary model of analysis since we anticipated variability in the design and population characteristics of the included trials. Results for a fixed-effects model (inverse-variance method) will be presented simultaneously as a sensitivity analysis.

#### Statistical heterogeneity

We will test for the presence of statistical heterogeneity across trial estimates using Cochran’s Q test [39] and the magnitude of the between-trial heterogeneity will be quantified with the I^2^ metric [40]. When feasible (i.e., 10 or more trials), we will investigate potential sources of statistical heterogeneity with the random-effects meta-regression analysis and subgroups analysis. Explanatory variables to be included in meta-regression models are described below.

#### Small-study and publication biases

We will investigate the association between trial size (precision) and treatment effects in contour-enhanced funnel plots, contrasting the effect estimates on the horizontal axis against their standard errors on the vertical axis, accompanied by a regression test for asymmetry. Furthermore, for continuous outcomes, small-study biases will be investigated by Egger’s regression test, whereas for binary outcomes we will use Harbord’s test [41].

### Equivalence testing

#### Criteria to claim equivalence

**Fig. 1:**
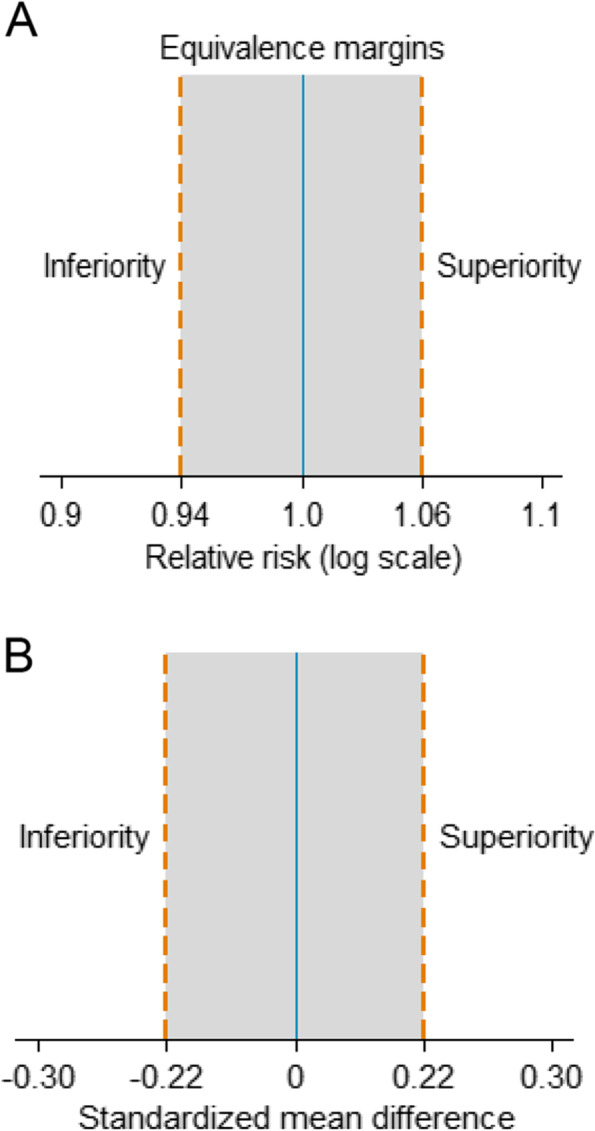
Boundaries of equivalence (dashed lines) for a two-sided 95% confidence interval of the treatment difference. **A** Equivalence margins for ACR20 (primary outcome). **B** Equivalence margins for HAQ-DI (co-primary outcome). If the summary 95% CI lies within the gray regions, the null hypothesis will be rejected, and equivalence will be claimed

Equivalence will be evaluated and interpreted using predefined margins of equivalence (Fig. 1). Upper and lower equivalence bounds were specified based on the smallest effect size of clinical importance. These values were computed from large placebo-controlled trials and validated by two rheumatologists with extensive experience treating patients with RA. Prespecified boundaries of equivalence will be applied to the primary and co-primary outcomes only.

Based on random-effects models, lower and upper confidence limits will be calculated. For a specific outcome (i.e., either ACR20 or HAQ-DI), if the two-sided 95% confidence interval for the difference in effect is completely contained within the prespecified boundaries of equivalence, biosimilars and biologics will be considered equivalent. However, the rejection of non-equivalence (for both ACR20 and HAQ-DI outcomes) will be required for biosimilars to be declared overall equivalent to biologic drugs. Secondary outcomes will be examined through standard superiority tests (two-tailed).

#### Margins of equivalence: ACR20 criteria

For the ACR20 outcome, we prespecified equivalence margins to preserve 90% of the effects observed with biologics using the fixed-margin method [42]. Specifically, we calculated the equivalence margins as:

1 $$Equivalence \ margins{\text{ = }}e^{{ \pm m}}$$

with

2 $$m = log\left( {RR^{{PE - 1}} } \right)$$

where PE stands for the preserved effect (range 0 to 1 [100%]). Based on a network meta-analysis by Guyott et al. [43] that included 11 randomized trials with 3762 patients who were unresponsive to methotrexate, the RR under a random-effects model for ACR20 at 6 months for any biologics (adalimumab/etanercept/infliximab) vs. placebo was approximately 1.80. Similar estimates were obtained considering a combination of both methotrexate-naïve and methotrexate unresponsive patients, in which the frequentist summary RR of achieving ACR20 was 1.81 (random-effects model, 13 trials, 7087 patients) for the comparison adalimumab/etanercept/infliximab vs. placebo [44]. Thus, biosimilars will be considered equivalent to biologics if the 95% confidence limits for the summary RR lie within the 0.94 and 1.06 interval (Fig. 1A).

#### Margins of equivalence: HAQ-DI

For HAQ-DI, which is a continuous outcome (with higher scores meaning worse function status), equivalence margins were constructed under the clinical assumption that an increase equal or larger than 0.15 units over 1 year on the 0-to-3 HAQ-DI scale is considered clinically perceptible by the patients [45]. Based on trials encompassing a diversified group of RA patients that received adalimumab, etanercept, or infliximab (1816 patients) [46–48], we estimated the HAQ-DI population standard deviation after a 24/26-week treatment to be approximately 0.69. Therefore, on a standardized mean difference scale, the 0.15 units difference is approximately equivalent to ± 0.22 standard deviations (Fig. 1B).

### Analysis of subgroups and meta-regression

We will perform prespecified subgroup analyses. When feasible, the following subgroup analysis will be conducted:

- Type of molecule (infliximab vs. etanercept vs. adalimumab);
- Concomitant use of synthetic disease-modifying antirheumatic drugs (yes or no);
- Sample size (average of  > 100 patients per group vs. < 100 patients per group);
- Allocation concealment (low risk vs. high risk/unclear risk);
- Trial duration (equivalence studies) (3 vs. 6 vs. 12 months);
- Type of design (multiple switching/switching studies vs. transitional studies)
- Funding independent of industry (yes vs. no/unclear); and
- Publication status (published vs. unpublished).

The above-mentioned variables will also serve as explanatory variables in meta-regression models.

### Sensitivity analysis (primary and co-primary outcomes)

#### Non-inferiority

As there would be minor concerns if biosimilar drugs were more efficacious than conventional biologicals, exploratory non-inferiority analysis will be conducted in the event the data do not support the equivalence. Specifically, we will claim non-inferiority if the lower limit of the 95% CI is above the prespecified cutoffs for both ACR20 and HAQ-DI.

#### Time points

Sensitivity analyses for the primary and co-primary outcomes will also be performed at different time points: 1, 3, 8, 12, 36, and 48 months. All these analyses will be considered exploratory and will be conducted from the superiority testing point of view.

### Assessment of overall certainty of evidence

The overall certainty of evidence will be assessed by two investigators and will be based on the Grading of Recommendations Assessment, Development and Evaluation (GRADE) system [49]. Assessments will be conducted by outcome. Disagreements will be settled by consensus or discussion with a third reviewer. The certainty of evidence of each outcome will be graded as very low, low, moderate, or high. The following domains will be assessed:

- Study design and risk of bias
- Inconsistency
- Indirectness
- Imprecision
- Other factors (e.g., reporting bias, publication bias)

#### Ranking of outcomes by their relative importance

We have adopted the recommendations from the GRADE handbook for selecting and rating the importance of outcomes [27]. Specifically, we ranked each outcome as “critical,” “important but not critical,” and “limited importance to decision-making” (Table 2).

**Table 2:** Ranking of outcomes

**Outcome**	**Rating scale 1–9**^**a**^** (importance of the outcome for decision-making)**	**Type of outcome**
**Disease activity/clinical response**		
CDAI	9 (critical)	Continuous or binary
DAS-28 ESR	8.5 (critical)	Continuous or binary
SDAI	8.5 (critical)	Continuous or binary
ACR 20	8 (critical)	Binary
ACR 50	8 (critical)	Binary
DAS-28 CRP	8 (critical)	Continuous or binary
ACR-N	7.5 (critical)	Continuous
ACR 70	6 (important but not critical)	Binary
EULAR response	5 (important but not critical)	Binary
**Safety**		
TEAE	9 (critical)	Binary
Serious TEAE	9 (critical)	Binary
Death related to treatment	9 (critical)	Binary
IRRs	9 (critical)	Binary
Hypersensitivity	9 (critical)	Binary
Active tuberculosis	9 (critical)	Binary
Serious infections	9 (critical)	Binary
Death all causes	8 (critical)	Binary
Overall discontinuation rate	8.5 (critical)	Binary
Malignancies	8 (critical)	Binary
Fatigue	7 (critical)	Binary
**Function capacity/quality of life**		
HAQ-DI	8.5 (critical)	Continuous
SF-36 score—physical component summary	8 (critical)	Continuous
SF-36 score- mental component summary	8 (critical)	Continuous
**Structural damage progression**		
mTRSS or Sharp/van der Heijde score	5.5 (important but not critical)	Continuous
**Immunogenicity**		
Incidence of ADAs	5 (important but not critical)	Binary
NABs—positive	5 (important but not critical)	Binary

*ACR* The American College of Rheumatology, *CRP* C-reactive protein level, *HAQ-DI* Health Assessment Questionnaire—Disability Index, *VAS* Visual analog scale, *SDAI* Simplified Disease Activity Score, *DA* disease activity, *CDAI* Clinical Disease Activity Score, *DAS28-ESR* Disease Activity Score in 28 joints based on the erythrocyte sedimentation rate, *DAS28-CRP* Disease Activity Score in 28 joints, four components based on C-reactive protein, *ACR-N* The numeric index of the ACR response, *EULAR* European League Against Rheumatism, *SF-36* The Medical Outcomes Study 36-item Short-Form Health Survey, *mTRSS* Sharp/van der Heijde score, *IRRs* Infusion-related reactions, *TEAE* Overall treatment emergent adverse event

^a^Based on the mean average ranking of two rheumatologists with extensive clinical experience in treating RA patients and a physical therapist with advanced training in evidence synthesis

The ranking was conducted through consultations with two clinical specialists (rheumatologists) and a physical therapist specialized in evidence synthesis. These professionals were invited to participate based on their clinical experience, academic background, and the lack of any conflict of interest. Briefly, before the scoping meeting, based on previous systematic reviews, we screened the list of outcomes (both primary and secondary outcomes) of 14 trials that we knew a priori that met all the eligibility criteria. Subsequently, we created an integrated list of all outcomes and categorized them into five main domains:

- Disease activity/clinical response
- Function capacity/quality of life
- Structural damage progression
- Immunogenicity
- Safety

Through an iterative approach in a single scoping meeting, each member of the collaborative working group ranked outcomes independently. Conflicting ranking cases were discussed jointly until a consensus was reached.

## Discussion

At the time of writing this manuscript, more than 16 biosimilars of adalimumab, etanercept, and infliximab had been approved in the USA, Europe, Canada, and Latin America for the treatment of RA [50–53]. The proposed systematic review will comprehensively assess the efficacy, safety, and immunogenicity of these biosimilars compared to their originator molecules and examine the clinical consequences of switching from biologics to biosimilars in the management of RA patients.

The systematic review described here, to the best of our knowledge, is the first one proposing a comprehensive, clinician-oriented approach to assess the equivalence and the impact of switching between biosimilars and biologics on the management of patients with RA. Over the past years, relevant systematic reviews on the differences between biosimilar drugs vs. reference molecules in rheumatoid arthritis have been published [54–57]. However, only a few of the previous systematic reviews considered all pertinent domains of risk of bias that are specific to biosimilar drugs [57]. Similarly, only a few reviews graded the certainty of evidence according to the GRADE system [57]. Besides, we propose the first systematic evaluations based on equivalence testing at the level of meta-analysis using equivalence margins elicited from clinical specialists. We believe that our systematic review will add to the body of evidence in biosimilar drugs, building confidence for patients and clinicians, and providing healthcare systems with updated information that can help in their decision-making processes.

Furthermore, by examining the randomized evidence on the effects of a wide range of biosimilars, we will also be able to address whether switching from reference biologics to biosimilars or vice and versa, in general, results in similar clinical benefits with acceptable immunogenicity and safety profiles. As a result, our findings hold great potential to affect not only the therapeutic regimen of RA patients that will use a DMARD for the first time but also the treatment of those that will have their treatment substituted from a biologic to a biosimilar, or from a biosimilar to a biologic.

Overall, we expect that our results will guide clinicians, researchers, decision-makers, stakeholders, and policymakers about the efficacy, safety, immunogenicity, and substitution and interchangeability of currently marketed biosimilars for the treatment of RA patients and assist healthcare systems to employ more efficiently the scarce existing resources.

## Supplementary Information

**Additional file 1.** PRISMA for systematic review protocols (PRISMA-P) Checklist. Source: Moher et al. [21].

**Additional file 2.** Search Strategies of electronic database and other sources.

**Additional file 3.** Secondary outcomes of efficacy (disease activity measures, functional capacity, quality of life and structural damage progression) and safety. ACR: the American College of Rheumatology; CRP: C-Reactive Protein level; VAS: visual analog scale; HAQ-DI: Health Assessment Questionnaire - Disability Index; VAS: visual analog scale; SDAI: Simplified Disease Activity Score DA: disease activity; CDAI: Clinical Disease Activity Score DAS28-ESR: Disease Activity Score in 28 joints based on the erythrocyte sedimentation rate; DAS28-CRP: Disease Activity Score in 28 joints, four components based on C-reactive protein; SJC: Swollen joint count; TJC: Tender Joint Count; ACR-N: The numeric index of the ACR response; EULAR: European League Against Rheumatism; SF-36: The Medical Outcomes Study 36-item Short-Form Health Survey; mTRSS: Sharp/van der Heijde score; TEAE: Overall Treatment Emergent Adverse Event; infusion-related reactions.

**Additional file 4.** Criteria to identify bias on equivalence or non-inferiority studies. Sources: Cochrane Risk of Bias in randomized trials (ROB 1.0.) [36] and the US Agency for Healthcare Research and Quality recommendations [24].

**Additional file 5.** Criteria to identify bias on switching studies. Notes: * It must be clearly pointed out. The wash-out period is defined as the time between the discontinuation of one biologic and the initiation of a second biologic. This wash-out period is arbitrarily based on the half-life of the biologic, namely the time needed to eliminate 50% of the biologic from the bloodstream.; ** The comparative assessment should occur during the final exposure period after enough time (i.e., an adequate washout period of at least three or more half-lives) has elapsed following the last administration of the reference product in the switching arm; The number of doses of the proposed interchangeable product or reference product administered in the final exposure period will depend on the half-life and clinical dosing regimen.; The serum half-time of infliximab is around 14 days or 2 weeks; Etanercept has a mean ± standard deviation half-life of 102 ± 30 hours was observed ( more or less 4 days); The mean terminal half-life of adalimumab was approximately 2 weeks. Sources: Moots et al. [28] and FDA [14].

## Abbreviations

RA: Rheumatoid arthritis
DMARDs: Disease-modifying antirheumatic drugs
FDA: The US Food and Drug Administration
PRISMA-P: The Preferred Reporting Items for Systematic Reviews and Meta-Analyses Protocols guidelines
PROSPERO: The International Prospective Register of Systematic Reviews
PRISMA: Preferred Reporting Items for Systematic Review and Meta-analysis
US: United States
EU: European
CENTRAL: Cochrane Central Register of Controlled Trials
LILACS: Latin American and Caribbean Health Science
RCTs: Randomized controlled trials
ACR20: The American College of Rheumatology 20 criteria
ACR 50: The American College of Rheumatology 50 criteria
ACR70: The American College of Rheumatology 70 criteria
VAS: Visual analog scale
HAQ-DI: Health Assessment Questionnaire—Disability Index
DAS28-ESR: Disease activity score in 28 joints based on the erythrocyte sedimentation rate
DAS28-CRP: Disease activity score in 28 joints with four components based on C-reactive protein
ACR-N: The numeric index of the ACR response
SF-36: The Medical Outcomes Study 36-item Short-Form Health Survey
mTRSS: Sharp-Van Der Heidje Modified Score Method
TEAEs: Treatment-emergent adverse events
IRRs: Infusion-related reactions
ADAs: Anti-drug antibodies
Nabs: Neutralizing antibodies
PP: Per-protocol
ITT: Intention-to-treat
RR: Relative risk
SMD: The standardized mean difference
PE: Preserve effect
Log: Logarithm
GRADE: The Grading of Recommendations Assessment, Development and Evaluation System
