# Supplementary material for: Equivalence and switching between biosimilars and reference molecules in rheumatoid arthritis: protocol for a systematic review and meta-analysis
Source: Syst Rev. 2021 Jul 17;10:205. doi: 10.1186/s13643-021-01754-x (PMC8286602; doi:10.1186/s13643-021-01754-x)
Supplement: Supplementary file 2 — Additional file 2. Search Strategies of electronic database and other sources. [file 13643_2021_1754_MOESM2_ESM.docx]

| **Database/Biologic** | **Search Strategy** |
| --- | --- |
| **ELETRONIC DATABASES** | |
| PubMed -etanercept | ((((((((("Arthritis, Rheumatoid"[Mesh]) OR Rheumatoid Arthritis)) OR ((((("Arthritis, Juvenile"[Mesh]) OR "Rheumatoid Arthritis, Systemic Juvenile" [Supplementary Concept]) OR Juvenile Arthritis) OR Arthritis, Juvenile Chronic) OR Arthritis, Juvenile Idiopathic)))))) AND ((((((((((("Biosimilar Pharmaceuticals"[Mesh]) OR Pharmaceuticals, Biosimilar) OR Follow-on Biologics) OR Biologics, Follow-on) OR Follow on Biologics) OR Subsequent Entry Biologics) OR Biologics, Subsequent Entry) OR Biosimilars)))) AND (((((((((("Etanercept"[Mesh]) OR TNFR-Fc Fusion Protein) OR Recombinant Human Dimeric TNF Receptor Type II IgG Fusion Protein) OR TNF Receptor Type II IgG Fusion Protein) OR Erelzi) OR ("GP2015" [Supplementary Concept] OR "LBEC0101" [Supplementary Concept]))) OR benepali) OR etanercept szzs)) |
| PubMed - infliximab | (((((((((((("Arthritis, Rheumatoid"[Mesh]) OR Rheumatoid Arthritis)) OR ((((("Arthritis, Juvenile"[Mesh]) OR "Rheumatoid Arthritis, Systemic Juvenile" [Supplementary Concept]) OR Juvenile Arthritis) OR Arthritis, Juvenile Chronic) OR Arthritis, Juvenile Idiopathic))))))))) AND ((((((((((((((("Biosimilar Pharmaceuticals"[Mesh]) OR Pharmaceuticals, Biosimilar) OR Follow-on Biologics) OR Biologics, Follow-on) OR Follow on Biologics) OR Subsequent Entry Biologics) OR Biologics, Subsequent Entry) OR Biosimilars))))))) AND (((((((("Infliximab"[Mesh])) OR "SB2 infliximab" [Supplementary Concept]) OR "GP1111" [Supplementary Concept])) OR "CT-P13" [Supplementary Concept])) OR (Infliximab-abda[Text Word]) OR Infliximab-dyyb[Text Word]) OR Infliximab-qbtx[Text Word]) OR Infliximab-axxq[Text Word]) OR Inflectra[Text Word]) OR Renflexis[Text Word]) OR Ixifi[Text Word]) OR Zessly[Text Word]) OR Flixabi) OR Remsima[Text Word]))) |
| PubMed - adalimumab | (((((((((((("Arthritis, Rheumatoid"[Mesh]) OR Rheumatoid Arthritis)) OR ((((("Arthritis, Juvenile"[Mesh]) OR "Rheumatoid Arthritis, Systemic Juvenile" [Supplementary Concept]) OR Juvenile Arthritis) OR Arthritis, Juvenile Chronic) OR Arthritis, Juvenile Idiopathic))))))))) AND ((((((((((((((("Biosimilar Pharmaceuticals"[Mesh]) OR Pharmaceuticals, Biosimilar) OR Follow-on Biologics) OR Biologics, Follow-on) OR Follow on Biologics) OR Subsequent Entry Biologics) OR Biologics, Subsequent Entry) OR Biosimilars))))))) AND ((((((((((((((((((((("Adalimumab"[Mesh]) OR D2E7 Antibody) OR Antibody, D2E7) OR Adalimumab-adbm) OR Adalimumab-atto) OR Adalimumab-adaz[Text Word]) OR Adalimumab-bwwd[Text Word]) OR Adalimumab-afzb[Text Word]) OR Amjevita[Text Word]) OR Hadlima[Text Word]) OR Cyltezo[Text Word]) OR Hyrimoz[Text Word]) OR Abrilada[Text Word]) OR Halimatoz[Text Word]) OR Hefiya[Text Word]) OR Imraldi[Text Word]) OR Hulio[Text Word]) OR Kromeya[Text Word]) OR Idacio[Text Word])) OR (((("ABP 501" [Supplementary Concept]) OR "GP2017" [Supplementary Concept]) OR "BI 695501" [Supplementary Concept]) OR "PF-06410293" [Supplementary Concept])) |
| EMBASE - etarnercept | ('rheumatoid arthritis'/exp OR 'rheumatoid arthritis' OR 'juvenile rheumatoid arthritis'/exp OR 'juvenile rheumatoid arthritis') AND ('biosimilar agent'/exp OR 'biosimilar agent' OR (('biosimilar' OR 'biosimilar'/exp OR biosimilar) AND pharmaceuticals) OR ('follow on' AND ('biologics' OR 'biologics'/exp OR biologics)) OR (subsequent AND entry AND ('biologics' OR 'biologics'/exp OR biologics))) AND ('etanercept'/exp OR 'etanercept' OR 'etanercept szzs'/exp OR 'etanercept szzs' OR 'erelzi' OR 'erelzi'/exp OR erelzi OR 'benepali' OR 'benepali'/exp OR benepali OR sb4 OR 'gp2015'/exp OR gp2015) AND [embase]/lim |
| EMBASE - infliximab | ('rheumatoid arthritis'/exp OR 'rheumatoid arthritis' OR 'juvenile rheumatoid arthritis'/exp OR 'juvenile rheumatoid arthritis') AND ('biosimilar agent'/exp OR 'biosimilar agent' OR (('biosimilar' OR 'biosimilar'/exp OR biosimilar) AND pharmaceuticals) OR ('follow on' AND ('biologics' OR 'biologics'/exp OR biologics)) OR (subsequent AND entry AND ('biologics' OR 'biologics'/exp OR biologics))) AND ('infliximab'/exp OR 'infliximab' OR 'inflectra' OR 'inflectra'/exp OR inflectra OR 'ixifi' OR 'ixifi'/exp OR ixifi OR 'renflexis' OR avsola OR 'zessly'/exp OR zessly OR 'remsima'/exp OR remsima OR 'renflexis'/exp OR renflexis OR 'infliximab dyyb'/exp OR 'infliximab dyyb' OR 'infliximab qbtx'/exp OR 'infliximab qbtx' OR 'infliximab axxq' OR 'infliximab abda'/exp OR 'infliximab abda' OR ctp13 OR sb2 OR 'abp710'/exp OR abp710 OR 'gp1111'/exp OR gp1111 OR 'pf06438179'/exp OR pf06438179) AND [embase]/lim |
| EMBASE - adalimumab | (('rheumatoid arthritis'/exp OR 'rheumatoid arthritis' OR 'juvenile rheumatoid arthritis'/exp OR 'juvenile rheumatoid arthritis') AND ('biosimilar agent'/exp OR 'biosimilar agent' OR (('biosimilar' OR 'biosimilar'/exp OR biosimilar) AND pharmaceuticals) OR ('follow on' AND ('biologics' OR 'biologics'/exp OR biologics)) OR (subsequent AND entry AND ('biologics' OR 'biologics'/exp OR biologics))) AND ('adalimumab'/exp OR 'adalimumab' OR 'amjevita' OR 'amjevita'/exp OR amjevita OR 'cyltezo' OR 'cyltezo'/exp OR cyltezo OR hadlima OR 'hyrimoz' OR 'hyrimoz'/exp OR hyrimoz OR 'adalimumab atto'/exp OR hefiya OR imraldi OR hulio OR kromeya OR idacio OR abrilada OR 'adalimumab atto' OR 'adalimumab adbm'/exp OR 'adalimumab adbm' OR 'adalimumab adaz'/exp OR 'adalimumab adaz') OR 'adalimumab afzb' OR abp501 OR gp2017 OR sb5 OR fkb327 OR msb11022 OR pf06410293) AND [embase]/lim |
| CENTRAL - etarnercept | #1 MeSH descriptor: [Biosimilar Pharmaceuticals] explode all trees  #2 MeSH descriptor: [Etanercept] explode all trees  #3 #1 AND #2 |
| CENTRAL - infliximab | #1 MeSH descriptor: [Biosimilar Pharmaceuticals] explode all trees  #2 MeSH descriptor: [Infliximab] explode all trees  #3 #1 AND #2 |
| CENTRAL - adalimumab | #1 MeSH descriptor: [Biosimilar Pharmaceuticals] explode all trees  #2 MeSH descriptor: [Adalimumab] explode all trees  #3 #1 AND #2 |
| LILACS – etarnercept | biosimilar [Palavras] and etanercept [Palavras] |
| LILACS - infliximab | biosimilar [Palavras] and infliximab [Palavras] |
| LILACS - adalimumab | biosimilar [Palavras] and adalimumab [Palavras] |
| **TRIAL DATABASES** | |
| the EU Clinical Trial Register - etanercept | biosimilar AND etanercept AND rheumatoid arthritis |
| the EU Clinical Trial Register - infliximab | biosimilar AND infliximab AND rheumatoid arthritis |
| the EU Clinical Trial Register - adalimumab | biosimilar AND adalimumab AND rheumatoid arthritis |
| Clinicaltrials - etanercept | BIOSIMILAR \| Etanercept \| rheumatoid arthritis |
| Clinicaltrials - infliximab | BIOSIMILAR \| Infliximab \| rheumatoid arthritis |
| Clinicaltrials - adalimumab | BIOSIMILAR \| Adalimumab \| rheumatoid arthritis |
| International Clinical Trials Registry Platform-World Health Organization - etanercept | biosimilar AND etanercept AND rheumatoid arthritis |
| International Clinical Trials Registry Platform-World Health Organization - infliximab | biosimilar AND infliximab AND rheumatoid arthritis |
| International Clinical Trials Registry Platform-World Health Organization - adalimumab | biosimilar AND adalimumab AND rheumatoid arthritis |
