# Supplementary material for: Equivalence and switching between biosimilars and reference molecules in rheumatoid arthritis: protocol for a systematic review and meta-analysis
Source: Syst Rev. 2021 Jul 17;10:205. doi: 10.1186/s13643-021-01754-x (PMC8286602; doi:10.1186/s13643-021-01754-x)
Supplement: Supplementary file 3 — Additional file 3. Secondary outcomes of efficacy (disease activity measures, functional capacity, quality of life and structural damage progression) and safety. ACR: the American College of Rheumatology; CRP: C-Reactive Protein level; VAS: visual analog scale; HAQ-DI: Health Assessment Questionnaire - Disability Index; VAS: visual analog scale; SDAI: Simplified Disease Activity Score DA: disease activity; CDAI: Clinical Disease Activity Score DAS28-ESR: Disease Activity Score in 28 joints based on the erythrocyte sedimentation rate; DAS28-CRP: Disease Activity Score in 28 joints, four components based on C-reactive protein; SJC: Swollen joint count; TJC: Tender Joint Count; ACR-N: The numeric index of the ACR response; EULAR: European League Against Rheumatism; SF-36: The Medical Outcomes Study 36-item Short-Form Health Survey; mTRSS: Sharp/van der Heijde score; TEAE: Overall Treatment Emergent Adverse Event; infusion-related reactions. [file 13643_2021_1754_MOESM3_ESM.docx]

| **Outcomes** | **Description** | **Type of variable/ Type of outcome/ Extraction** |
| --- | --- | --- |
| **Disease activity** | | |
| ACR response: ACR20 | A participant was a responder if the following 3 criteria for improvement from Baseline were met:  ≥ 20% improvement in TJC; ≥ 20% improvement in SJC; and ≥ 20% improvement in at least 3 of the 5 following parameters: Patient's assessment of pain (VAS 0 a 100 mm); Patient's global assessment of disease activity (Likert scale from 0 to 10); Physician's global assessment of disease activity (Likert scale from 0 to 10); Patient's self-assessment of physical function (HAQ-DI); CRP level. | Integer/  Composite and binary/  n participants with the event; n total of participants |
| ACR response: ACR50 | A participant was a responder if the following 3 criteria for improvement  from Baseline were met:  ≥ 50% improvement in TJC; ≥ 50% improvement in SJC; and ≥ 50% improvement in at least 3 of the 5 following parameters: Patient's assessment of pain (VAS 0 a 100 mm); Patient's global assessment of disease activity (Likert scale from 0 to 10); Physician's global assessment of disease activity (Likert scale from 0 to 10); Patient's self-assessment of physical function (HAQ-DI); CRP level. | Integer/  Composite and binary/  n participants with the event; n total of participants |
| ACR response: ACR70 | A participant was a responder if the following 3 criteria for improvement from Baseline were met: ≥ 70% improvement in TJC; ≥ 70% improvement in SJC; and ≥ 70% improvement in at least 3 of the 5 following parameters: Patient's assessment of pain (VAS 0- 100 mm); Patient's global assessment of disease activity (Likert scale from 0 to 10); Physician's global assessment of disease activity (Likert scale from 0 to 10); Patient's self-assessment of physical function (HAQ-DI); CRP level | Integer/  Composite and binary outcome/ n participants with the event; n total of participants |
| SDAI | Number of SJC (0-28); Number of TJC (0-28); CRP in mg/mL (0.1-10.0); Patient’s global disease activity assessment (VAS in cm 0-10); Patient’s global health assessment (VAS in cm 0-10); Physician's global assessment of disease activity (VAS in cm 0-10).  Total index: 0.1-86  Remission (≤ 3.3);  Low DA (<3.3 and ≤ 11);  Moderate DA (<11 and ≤ 26);  High disease activity (<26). | Integer or float/  Composite and categorical or continuous/  Categoric outcome: n participants with remission; n participants with low DA; n participants with moderate DA; n participants with high DA; total of participants/  Continuous outcome: mean, SD, median, IQR 25% and 75%, minimum and maximum, n total participants - values will be extract from baseline, follow up and change from baseline |
| CDAI | Number of SJC (0-28); Number of TJC (0-28); Patient’s global disease activity assessment (VAS in cm 0-10); Physician's global assessment of disease activity (VAS in cm 0-10).  Total index: 0-76  Remission (≤ 2.8);  Low DA (<2.8 and ≤ 10);  Moderate DA (<10 and ≤ 22);  High DA (<22). | Integer or float/  Composite and categorical or continuous/  Categorical outcome: n participants with remission; n participants with low DA; n participants with moderate DA; n participants with high DA; total of participants/  Continuous outcome: mean, SD, median, IQR 25% and 75%, minimum and maximum, n total participants - values will be extract from baseline, follow up and change from baseline |
| DAS28- ESR | Number of SJC, square root transformed (0-1.48); Number of TJC, square root transformed (0-2.96); ESR, log transformed (0.49-3.22); Patient’s global health assessment (VAS in cm 0-10).  Total index (0.49-9.07);  Remission (≤ 2.6);  Low DA (<2.6 and ≤ 3.2);  Moderate DA (<3.2 and ≤ 5.1);  High DA (<5.1). | Integer or float/  Composite and categorical or continuous/  Categorical outcome: n participants with remission; n participants with low DA; n participants with moderate DA; n participants with high DA; total of participants/  Continuous outcome: mean, SD, median, IQR 25% and 75%, minimum and maximum, n total participants - values will be extract from baseline, follow up and change from baseline |
| DAS28- CRP | Number of SJC, square root transformed (0-1.48); Number of TJC, square root transformed (0-2.96); CRP level; Patient’s global health assessment (VAS in cm 0-10).  Total index (0.49-9.07).  Remission (≤ 2.6);  Low DA (<2.6 and ≤ 3.2);  Moderate DA (<3.2 and ≤ 5.1);  High DA (<5.1). | Integer or float/  Composite and categorical or continuous/  Categorical outcome: n participants with remission; n participants with low DA; n participants with moderate DA; n participants with high DA; total of participants/  Continuous outcome: mean, SD, median, IQR 25% and 75%, minimum and maximum, n total participants - values will be extract from baseline, follow up and change from baseline |
| ACR-N | Provides a single number that characterizes  the percentage of improvement from baseline that a patient has experienced in analogy to ACR20, ACR50, and ACR70 responses. A median ACR-N of 42, for example, would mean that the patient with the median ACR-N value experienced an improvement of 42%.  Total index (0-100) | Float/  Continuous /  Mean, SD, SE, median, IQR 25% and 75%, minimum and maximum, n total participants - values will be extract from baseline, follow up and change from baseline |
| EULAR-ESR | Classify patients as good, moderate, or non-responders, using the individual amount of change in the DAS28-ESR and the DAS28-ESR value (low, moderate, or high) reached.  No response - Improvement in DAS28 from baseline ≤ 0.6.  Moderate response - Improvement in DAS28 from baseline >0.6 and ≤ 1.2.  Good response - Improvement in DAS or DAS28 from baseline ≤ 1.2. | Integer /  Composite and categorical outcome: n participants with remission; n participants with good response; n participants with moderate response; n participants with no response; total of participants/  Continuous outcome: mean, SD, SE, median, IQR 25% and 75%, minimum and maximum, n total participants - values will be extract from baseline, follow up and change from baseline |
| **Functional status** | | |
| HAQDI | It assesses the functional status of patients through the evaluation of eight domains of daily-life activities. (dressing and grooming, arising, eating, walking, hygiene, reach, grip, and activities) with 20 questions in total. For each question, there are four possible responses: 0 = without difﬁculty, 1 = with some difﬁculty, 2 = with much difﬁculty, and 3 = unable to do. The highest score reported for any component question in each domain determines the final score for that domain. By convention, the overall disability index is expressed on a 0 to 3 scale, representing an average score across the domains. A HAQ-DI of 0 indicates no functional disability, whereas a HAQ-DI of 3 denotes severe functional disability. | Integer /  Continuous outcome: mean, SD, SE, median, IQR 25% and 75%, minimum and maximum, n total participants - values will be extract from baseline, follow up and change from baseline |
| **Structural damage progression** | | |
| mTRSS | Sixteen areas for erosions on each hand and wrist, and six joints for each foot were considered. The erosion is evaluated from 0 to 5 points (1=discrete changes, 2 to 3=greater changes; score >3 includes the size of the erosions). The score for erosion ranges from 0 to 160 in the hands and from 0 to 120 in the feet (the maximum erosion score for a joint in the foot is 10).  JSN is assessed in 15 joints for each hand and wrist, and six joints for each foot. The JSN is evaluated from 0 to 4 points (0=normal space, 1=suspect narrowing, 2=global narrowing<50% of original space, 3=global narrowing >50% of original space or subluxation and 4=articular ankylosis or total luxation). The score for JSN ranges from 0 to 120 in the hands and from 0 to 48 in the feet. (The hand score has greater weight because more joints are scored.).  Total mTRSS index: 0 to 448 | Float/Continuous outcome: mean, SD, SE, median, IQR 25% and 75%, minimum and maximum, n total participants - values will be extract from baseline, follow up and change from baseline |
| **Health related quality of Life** | | |
| SF-36 score - physical component summary | the SF-36 is composed of 8 multi-item scales (35 items) assessing physical function (10 items), role limitations due to physical health problems (4 items), bodily pain (2 items), general health (5 items), vitality (4 items), social functioning (2 items), role limitations due to emotional problems (3 items) and emotional well-being (5 items). These eight scales can be aggregated into two summary measures: The Physical (PCS) and Mental (MCS) Component Summary scores. The Physical Component Summary scores is calculated by positively weighting the 4 subscales in the physical domain (PF, RP, BP and GH) and the remaining psychological domain subscales negatively.  Total index: 0-100 | Float/Continuous outcome: mean, SD, SE, median, IQR 25% and 75%, minimum and maximum, n total participants - values will be extract from baseline, follow up and change from baseline |
| SF-36 score- mental component summary | The SF-36 is composed of 8 multi-item scales (35 items) assessing physical function (10 items), role limitations due to physical health problems (4 items), bodily pain (2 items), general health (5 items), vitality (4 items), social functioning (2 items), role limitations due to emotional problems (3 items) and emotional well-being (5 items). These eight scales can be aggregated into two summary measures: The Physical and Mental Component Summary scores. The Mental Component Summary scores is calculated by positively weighting the 4 mental domain subscales (MH, V, SF and RE), and negatively weighting the 4 physical domain subscales.  Total index: 0-100 | Float/Continuous outcome: mean, SD, SE, median, IQR 25% and 75%, minimum and maximum, n total participants - values will be extract from baseline, follow up and change from baseline |
| **Safety** | | |
| TEAE | Undesirable events that is not present prior to medical treatment, or an already present event that worsens either in intensity or frequency following the treatment. | Integer and binary/  n participants with the event; n total of participants |
| Serious TEAE | Undesirable events that is not present prior to medical treatment, or an already present event that worsens either in intensity or frequency following the treatment. The event is serious when the patient outcome is death, life-threatening, hospitalization (initial or prolonged), disability or permanent damage, congenital anomaly/birth defect or other serious (important medical events) | Integer and binary /  n participants with the event; n total of participants |
| Discontinuation rate | Overall number of patients that discontinued the treatment after enrolling the study | Integer and binary /  n participants with the event; n total of participants |
| Fatigue | An unfavorable feeling of fatigue temporally associated with the use of a medical treatment. We will extract the outcome rates as judged by study investigators. | Integer and binary /  n participants with the event; n total of participants |
| Injection site reactions (ISRs) | A local phenomenon defined as a constellation of subcutaneous symptoms, including swelling, erythema, pruritus, and pain around the site of injection. | Integer and binary /  n participants with the event; n total of participants |
| Infusion related reactions (IRRs) | Reactions experienced by patients during the infusion of monoclonal antibody therapy (uniphasic reaction) and/or within hours of an infusion (biphasic/delayed reaction). The reaction may be caused by the therapeutic agent, diluent, or delivery vehicle. Symptoms can include flushing, alterations in heart rate and blood pressure, dyspnea, bronchospasm, back pain, fever, urticaria, oedema, nausea and all types of rashes. | Integer and binary /  n participants with the event; n total of participants |
| Hypersensitivity | Hypersensitivity reactions are beta-type reactions. Clinical symptoms vary widely, from skin reactions to anaphylaxis. We will extract the outcome rates as judged by study investigators. | Integer and binary /  n participants with the event; n total of participants |
| Malignancies | A term for diseases in which abnormal cells divide without control and can invade nearby tissues. Malignant cells can also spread to other parts of the body through the blood and lymph systems. There are several main types of malignancy. Also called cancer. We will extract the outcome rates as judged by study investigators. | Integer and binary /  n participants with the event; n total of participants |
| Active tuberculosis | Active tuberculosis refers to disease that occurs in someone infected with Mycobacterium tuberculosis. It is characterized by signs or symptoms of active disease, or both, and is distinct from latent tuberculosis infection, which occurs without signs or symptoms of active disease. We will extract the outcome rates as judged by study investigators. | Integer and binary /  n participants with the event; n total of participants |
| Serious infections | Infections is the state produced by the establishment of one or more pathogenic agents (such as a bacteria, protozoans, or viruses) in or on the body of a suitable host. A serious infection as adverse event—that is, one needing hospitalization and/or intravenous antibiotic therapy, is life threatening or leading to persistent or significant disability. We will extract the outcome rates as judged by study investigators. | Integer and binary /  n participants with the event; n total of participants |
| Death all causes | All the deaths that occur in the study, regardless of the cause and treatment. We will extract the outcome rates as judged by study investigators. | Integer and binary /  n participants with the event; n total of participants |
| Death related to treatment | All the deaths that occur in the study related to the treatment. We will extract the outcome rates as judged by study investigators. | Integer and binary /  n participants with the event; n total of participants |
