# Supplementary material for: Equivalence and switching between biosimilars and reference molecules in rheumatoid arthritis: protocol for a systematic review and meta-analysis
Source: Syst Rev. 2021 Jul 17;10:205. doi: 10.1186/s13643-021-01754-x (PMC8286602; doi:10.1186/s13643-021-01754-x)
Supplement: Supplementary file 4 — Additional file 4. Criteria to identify bias on equivalence or non-inferiority studies. Sources: Cochrane Risk of Bias in randomized trials (ROB 1.0.) [36] and the US Agency for Healthcare Research and Quality recommendations [24]. [file 13643_2021_1754_MOESM4_ESM.docx]

| **Domains** | **Source** | **Criteria to identify bias** |
| --- | --- | --- |
| Random sequence generation | Cochrane RoB tool 1.0 | **Low risk of bias:** The investigators describe a random component in the sequence generation process such as:  *a) Referring to a random number table;  b)Using a computer random number generator;  c)Coin tossing; d) Shuffling cards or envelopes;  e) Throwing dice;  f)Drawing of lots;  g)Minimization** *Minimization may be implemented without a random element, and this is considered to be equivalent to being random.  **High risk of bias:** The investigators describe a non-random component in the sequence generation process. Usually, the description would involve some systematic, non-random approach, for example:  a) *The sequence generated by odd or even date of birth;  b) The sequence generated by some rule based on date (or day) of admission;  c) The sequence generated by some rule based on hospital or clinic record number.*  Other non-random approaches happen much less frequently than the systematic approaches mentioned above and tend to be obvious. They usually involve judgment or some method of non-random categorization of participants, for example:  *a) Allocation by the judgment of the clinician;  b) Allocation by the preference of the participant;  c) Allocation based on the results of a laboratory test or a series of tests;  d) Allocation by the availability of the intervention.*  **Unclear risk of bias:** Insufficient information about the sequence generation process to permit the judgment of ‘Low risk’ or ‘High risk’. |
| Allocation concealment | Cochrane RoB tool 1.0 | **Low risk of bias:** Participants and investigators enrolling participants could not foresee assignment because one of the following, or an equivalent method, was used to conceal allocation:  *a) Central allocation (including telephone, web-based and pharmacy-controlled randomization);  b) Sequentially numbered drug containers of identical appearance; c) Sequentially numbered, opaque, sealed envelopes.*  **High risk of bias:** Participants or investigators enrolling participants could possibly foresee assignments and thus introduce selection bias, such as allocation based on:  *a) Using an open random allocation schedule (e.g. a list of random numbers);  b) Assignment envelopes were used without appropriate safeguards (e.g. if envelopes were unsealed or non-opaque or not sequentially numbered);  c) Alternation or rotation; d) Date of birth;  e) Case record number;  f) Any other explicitly unconcealed procedure.*  **Unclear risk of bias:** Insufficient information to permit judgment of ‘Low risk’ or ‘High risk’. |
| Inconsistent application of inclusion/exclusion criteria | US Agency for Healthcare Research and Quality | **Low risk of bias:** The inclusion/exclusion criteria clearly stated and implemented consistently across all study participants.  Participants were not anticipated selected based on nonresponse or positive response Any or significant differences of baseline characteristics participants was detected   **High risk of bias:** There are differences between inclusion/exclusion criteria clearly stated and implemented consistently across all study participants. Participants in different arms were anticipated selected based on nonresponse or positive response  Examples:  *It was stated that participants to be included must not have taken drugs prior to biologics Disease-Modifying Antirheumatic Drugs (bDMARDs). However, different proportions of participants taking prior use of bDMARDs were found in the baseline.  Participants who have taken bDMARDs have the probability to have a better response*    **Unclear risk of bias:** Insufficient information to permit judgment of ‘Low risk’ or ‘High risk’. |
|  |  |  |
| Blinding of participants and investigators | US Agency for Healthcare Research and Quality | **Low risk of bias:** Any one of the following:  a*) No blinding or incomplete blinding, but the review authors judge that the outcome is not likely to be influenced by lack of blinding;  b) Blinding of participants and key study personnel ensured, and unlikely that the blinding could have been broken.*  **High risk of bias:** Any one of the following:  *a) No blinding or incomplete blinding, and the outcome is likely to be influenced by lack of blinding;  b) Blinding of key study participants and personnel attempted, but likely that the blinding could have been broken, and the outcome is likely to be influenced by lack of blinding.*    **Unclear risk of bias:** Insufficient information to permit judgment of ‘Low risk’ or ‘High risk’. |
| Participants behavior changes (poor adherence, use of concomitant treatments, and protocol violantions) | US Agency for Healthcare Research and Quality | **Low risk of bias:** The treatment adherence was sufficient in both of the study ‘s groups and across all subgroups. A high degree of patient adherence to treatment in both the new and active comparator groups The researchers stated any impact from a concurrent intervention or an unintended exposure that might bias or confound results; The same intended treatment regimen, from the schedule, and from the manner and precision of measuring outcomes between groups; The researchers stated any impact from a concurrent intervention or an unintended exposure that might bias or confound results  **High risk of bias:**  Any one of the following: *a) If adherence is poor within both treatment groups, the difference between groups would be underestimated. If adherence varies by treatment group, the difference between groups may be overestimated; b) The researchers did not rule out any impact from a concurrent intervention or an unintended exposure that might bias or confound results, and the concurrent intervention can bias the results; c) Deviations from the inclusion criteria, from the intended treatment regimen, from the schedule, and from the manner and precision of measuring outcomes;*  **Unclear risk of bias:**  Insufficient information to permit judgment of ‘Low risk’ or ‘High risk’; |
|  |  |  |
|  |  |  |
| Blinding of outcome assessors | Cochrane RoB tool 1.0 | **Low risk of bias:** Any one of the following:  a) *No blinding of outcome assessment, but the review authors judge that the outcome measurement is not likely to be influenced by lack of blinding;  b)Blinding of outcome assessment ensured, and unlikely that the blinding could have been broken.*  **High risk of bias:** Any one of the following:  a) *No blinding of outcome assessment and the outcome measurement is likely to be influenced by lack of blinding;  b) Blinding of outcome assessment, but likely that the blinding could have been broken, and the outcome measurement is likely to be influenced by lack of blinding.*  **Unclear risk of bias:**  Insufficient information to permit judgment of ‘Low risk’ or ‘High risk’;  The study did not address this outcome. |
|  |  |  |
| Outcomes measures |  | **Low risk of bias:** The outcome was measured using a validated instrument The outcome measure of interest is objective and it was objectively measured the outcome measured was properly administered (e.g., interviewers properly trained on interview protocol)  **High risk of bias:**  Any one of the following: a) *Use of nonvalid instruments or improper use of valid instruments to measure outcomes could lead to an underestimate or overestimate of the difference between groups. b) Use of a data collection method/mode that can influence the likelihood of outcome could mute or lead to an underestimate of the difference.*  **Unclear risk of bias:**  Insufficient information to permit judgment of ‘Low risk’ or ‘High risk’;  The study did not address this outcome. |
| Incomplete outcome data | Cochrane RoB tool 1.0 and US Agency for Healthcare Research and Quality | **Low risk of bias:** Any one of the following:  *a) No missing outcome data;*  *b) Reasons for missing outcome data unlikely to be related to true outcome (for survival data, censoring unlikely to be introducing bias);  c) Missing outcome data balanced in numbers across intervention groups, with similar reasons for missing data across groups;  d) For dichotomous outcome data, the proportion of missing outcomes compared with observed event risk not enough to have a clinically relevant impact on the intervention effect estimate;  f) For continuous outcome data, plausible effect size (difference in means or standardized difference in means) among missing outcomes not enough to have a clinically relevant impact on observed effect size;*  **g) *The study present both intention to treat (ITT) population analysis and results with dropouts excluded (or per-protocol (PP) analysis).*  High risk of bias:** Any one of the following:  *a) Reason for missing outcome data likely to be related to true outcome, with either imbalance in numbers or reasons for missing data across intervention groups;  b) For dichotomous outcome data, the proportion of missing outcomes compared with observed event risk enough to induce clinically relevant bias in intervention effect estimate; c) Missing data have been imputed using ONLY ITT analysis may underestimate the difference.* **Unclear risk of bias:** Any one of the following:  a) *Insufficient reporting of attrition/exclusions to permit judgement of risk’ of bias.*  *b) ‘Low risk’ or ‘High risk’ (e.g. number randomized not stated, no reasons for missing data provided).*  *c) The study did not address this outcome.* |
