# Supplementary material for: Equivalence and switching between biosimilars and reference molecules in rheumatoid arthritis: protocol for a systematic review and meta-analysis
Source: Syst Rev. 2021 Jul 17;10:205. doi: 10.1186/s13643-021-01754-x (PMC8286602; doi:10.1186/s13643-021-01754-x)
Supplement: Supplementary file 5 — Additional file 5. Criteria to identify bias on switching studies. Notes: * It must be clearly pointed out. The wash-out period is defined as the time between the discontinuation of one biologic and the initiation of a second biologic. This wash-out period is arbitrarily based on the half-life of the biologic, namely the time needed to eliminate 50% of the biologic from the bloodstream.; ** The comparative assessment should occur during the final exposure period after enough time (i.e., an adequate washout period of at least three or more half-lives) has elapsed following the last administration of the reference product in the switching arm; The number of doses of the proposed interchangeable product or reference product administered in the final exposure period will depend on the half-life and clinical dosing regimen.; The serum half-time of infliximab is around 14 days or 2 weeks; Etanercept has a mean ± standard deviation half-life of 102 ± 30 hours was observed ( more or less 4 days); The mean terminal half-life of adalimumab was approximately 2 weeks. Sources: Moots et al. [28] and FDA [14]. [file 13643_2021_1754_MOESM5_ESM.docx]

| **Specific domains for switching studies** | **Criteria to identify bias** | **Judgment** |
| --- | --- | --- |
| **Domain 1 – The randomized and blinded design with appropriate control arm** | There was a randomization step before the switch? | 1. Yes 2. No 3. Unclear |
|  | Did they keep the switching period blinded? | 1. Yes 2. No 3. Unclear |
|  | The study population was selected for a positive response or less disease severity? | 1. Yes 2. No 3. Unclear |
|  | What is your judgment about the randomized and blinded design with appropriate control arms? | **Low risk of bias:** There was a randomization step before the switch; They kept the switching period blinded; The study population was not selected for a positive response or less disease severity.  **High risk of bias:** There is no randomization step before the switch; It was an open-label period; The study population was selected for a positive response or less disease severity.  **Unclear risk of bias:** Insufficient information to permit judgment of ‘Low risk’ or ‘High risk’. |
| **Domain 2 – The number and way of switching** | The study had at least 1 arm incorporating switching between the proposed interchangeable product and the reference? | 1. Yes 2. No 3. Unclear |
|  | What is your judgment about the number and way of switching? | **Low risk of bias:** At least 1 arm incorporating switching between the proposed interchangeable product and the reference product, whereas the other arm remains on the reference product.  **High risk of bias:** It is not available 1 arm incorporating switching between the proposed interchangeable product and the reference product, whereas the other arm remains on the reference product.  **Unclear risk of bias:** Insufficient information to permit judgment of ‘Low risk’ or ‘High risk’. |
| **Domain 3 – The assessment of immunogenicity** | Immunogenicity was adequately measured in both switching and nonswitching arms? | 1. Yes 2. No 3. Unclear |
|  | Immunogenicity was measured for enough time (more than 12 months)? | 1. Yes 2. No 3. Unclear |
|  | What is your judgment about the assessment of immunogenicity? | **Low risk of bias:** Immunogenicity was adequately measured in both switching and nonswitching arms; Immunogenicity was measured for sufficient time (more than 12 months).  **High risk of bias:** Immunogenicity was not measured in both switching and nonswitching arms; Immunogenicity was not measured for sufficient time (less than 12 months).  **Unclear risk of bias:** Insufficient information to permit judgment of ‘Low risk’ or ‘High risk’. |
| **Domain 4 – The washout period between treatment** | Had the study a wash-period before the switch? * | 1. Yes 2. No 3. Unclear |
|  | If yes, the washout was done for enough time? ** | 1. Yes 2. No 3. Unclear |
|  | What is your judgment about the washout period between treatment (multiple switching)? | **Low risk of bias:** There is an enough and appropriate wash-out period before switching  **High risk of bias:** There is no wash-out period before switching  **Unclear risk of bias:** Insufficient information to permit judgment of ‘Low risk’ or ‘High risk’. |
| **Domain 5 – Enough power to assess efficacy and safety (equivalence studies)** | Had the study a small number (<50) of patients in the switch groups? | 1. Yes 2. No 3. Unclear |
|  | Was there a high rate of differential loss of participants before switching? | 1. Yes 2. No 3. Unclear |
|  | Was the study powered to assess efficacy in individual diseases? | 1. Yes 2. No 3. Unclear |
|  | What is your judgment about enough power to assess efficacy and safety (equivalence studies)? | **Low risk of bias:** Statistical power was enough, i.e. There are many patients in the switch groups.  There was a low or similar rate of loss of participants before switching;  The study was powered to assess efficacy in individual diseases.  **High risk of bias:** Statistical power was limited because of small patient numbers in the switch groups;  There was a high or differential loss of participants before switching; The study was NOT powered to assess efficacy in individual diseases.  **Unclear risk of bias:** Insufficient information to permit judgment of ‘Low risk’ or ‘High risk’. |
| **Domain 6 – An enough follow-up period** | The follow-up period after a switch was sufficiently long to allow detection of clinically relevant differences (equal or less than 24 weeks)? | 1. Yes 2. No 3. Unclear |
|  | What is your judgment about enough follow-up period? | **Low risk of bias:** The follow-up period after a switch was sufficiently long to allow the detection of clinically relevant differences ( More than 24 weeks)  **High risk of bias:** The follow-up period after a switch was not sufficiently long to allow detection of clinically relevant differences (equal or less than 24 weeks)  **Unclear risk of bias:** Insufficient information to permit judgment of ‘Low risk’ or ‘High risk’. |
